# Nobiletin Improves D-Galactose-Induced Aging Mice Skeletal Muscle Atrophy by Regulating Protein Homeostasis

**DOI:** 10.3390/nu15081801

**Published:** 2023-04-07

**Authors:** Hui-Hui Wang, Yan Zhang, Tai-Qi Qu, Xue-Qin Sang, Yi-Xuan Li, Fa-Zheng Ren, Peng-Cheng Wen, Ya-Nan Sun

**Affiliations:** 1 Key Laboratory of Precision Nutrition and Food Quality, Department of Nutrition and Health, China Agricultural University, Beijing 100083, China; 2 College of Food Science and Engineering, Gansu Agricultural University, Lanzhou 730070, China; 3 Food Laboratory of Zhongyuan, Luohe 462000, China

**Keywords:** aging, sarcopenia, protein homeostasis, nobiletin

## Abstract

Sarcopenia, a decrease in skeletal muscle mass and function caused by aging, impairs mobility, raises the risk of fractures, diabetes, and other illnesses, and severely affects a senior’s quality of life. Nobiletin (Nob), polymethoxyl flavonoid, has various biological effects, such as anti-diabetic, anti-atherogenic, anti-inflammatory, anti-oxidative, and anti-tumor properties. In this investigation, we hypothesized that Nob potentially regulates protein homeostasis to prevent and treat sarcopenia. To investigate whether Nob could block skeletal muscle atrophy and elucidate its underlying molecular mechanism, we used the D-galactose-induced (D-gal-induced) C57BL/6J mice for 10 weeks to establish a skeletal muscle atrophy model. The findings demonstrated that Nob increased body weight, hindlimb muscle mass, lean mass and improved the function of skeletal muscle in D-gal-induced aging mice. Nob improved myofiber sizes and increased skeletal muscle main proteins composition in D-gal-induced aging mice. Notably, Nob activated mTOR/Akt signaling to increase protein synthesis and inhibited FOXO3a-MAFbx/MuRF1 pathway and inflammatory cytokines, thereby reducing protein degradation in D-gal-induced aging mice. In conclusion, Nob attenuated D-gal-induced skeletal muscle atrophy. It is a promising candidate for preventing and treating age-associated atrophy of skeletal muscles.

## 1. Introduction

Skeletal muscles constitute approximately 40% of an adult’s body weight and are the most important motor organs [1,2]. Sarcopenia is an age-dependent decline in skeletal muscle mass and function in the elderly that is accompanied by increased morbidity, mortality, and a higher risk of falls and fractures [3]. Sarcopenia is a multifactorial disease associated with aging, physical inactivity, chronic inflammation, defective mitochondria, disrupted proteostasis, etc. [3,4]. Among them, skeletal muscle mass maintenance mainly depends on protein homeostasis, the overall balance between protein synthesis and degradation [5,6]. Myofibrillar proteins are the main protein that consists of skeletal muscle (55–60%), and these proteins are continuously synthesized and degraded [5,7]. Consequently, regulating protein synthesis and degradation is essential for preventing and curing skeletal muscle atrophy [5].

Sarcopenia cases continue to rise and have become increasingly prevalent due to the aging global population, which is now becoming a serious public health issue impacting about 15% of individuals aged 65 or over and accounts for about $18 billion annually in direct healthcare expenses in the United States alone [3,8]. Despite the presence of some nutritional and dietary approaches to prevent or treat sarcopenia, including protein, β-hydroxy β-methyl butyrate (HMB), and vitamin D, no effective therapies are approved for clinical use. Given the inconsistent results of previous studies on the benefits of protein supplementation on skeletal muscle mass and function in the elderly, it is still unclear if it is necessary or should be increased [9]. Notably, contradictory results have been reported in recent study on the effect of HMB on improving skeletal muscle mass and function in older individuals [10]. Although vitamin D supplementation has been shown to increase muscle mass in older individuals, the exact dosage, frequency, and length of treatment are unknown [9]. Therefore, finding safe, effective and affordable interventions to counteract mass loss is imperative. Nobiletin (Nob) is a natural polymethoxyl flavonoid extracted from citrus peels [11]. Nob has been widely reported for its numerous biological effects, such as anti-diabetic, anti-atherogenic, hepatic-protective, anti-tumor, anti-oxidant, anti-inflammatory functions, as well as for its maintenance of metabolic homeostasis and improvement of mitochondrial function [11,12,13,14]. Additionally, many anti-inflammatory drugs, metabolic agents, and hormones have been reported to have the potential to attenuate or prevent sarcopenia [15]. Some investigations reported that chronic inflammation usually increases with age, and inflammatory cytokines could activate protein degradation pathways and lead to muscle wasting [7,13,16]. Moreover, inflammatory factors could lead to insulin resistance and suppress the Insulin–Akt pathway, thereby affecting protein synthesis [17,18,19]. Notably, a previous study reported treatment of high-fat diet-fed rats with Nob markedly reduced insulin resistance [11]. Nohara et al. also discovered that Nob dramatically reduced high-fat diet-fed-induced increases in the levels of inflammation cytokines, such as lipopolysaccharide-binding peptide (LBP) and tumor necrosis factor-α (TNF-α) [12]. 

Therefore, we hypothesize that Nob can potentially maintain skeletal muscle mass by regulating protein synthesis and degradation. Based on previous investigations, we established a model of sarcopenia using D-gal induction to investigate whether Nob could counteract skeletal muscle atrophy during the aging process and to elucidate its underlying molecular mechanism. Overall, this work aimed to provide theoretical support for Nob’s advantageous effects on sarcopenia prevention and treatment.

## 2. Materials and Methods

### 2.1. Drugs and Reagents

Nobiletin (Nob, IN0210) was obtained from MedChemExpress (Beijing, China). Nob was prepared as a stock solution with DMSO, dispense and kept at −80 °C and use up within six months. D-galactose (D-gal, G5388) was purchased from Sigma-Aldrich (St. Louis, MO, USA). RIPA Lysis Buffer (P0013B), Phenylmethanesulfonyl fluoride (PMSF, ST506), 5× SDS-PAGE protein loading buffer (P0015L), BCA Protein Assay Kit (P0012), Mouse TNF-α ELISA Kit (PT512), and Mouse IL-6 ELISA Kit (PI326) were purchased from Beyotime Biotechnology Company (Shanghai, China). cDNA synthesis kit (G592) was obtained from abm (Vancouver, Canada). PowerUp™ SYBR™ Green (A25742) was purchased from Thermo Fisher Scientific (Waltham, MA, USA). Mouse LBP ELISA Kit (ab269542) was obtained from abcam (Shanghai, China). The primers were purchased from Beijing Tianyi Huiyuan Biotechnology Co., Ltd. (Beinjing, China). p-Ser2448-mTOR (5536T), p-p70 S6 Kinase Antibody (9234S) and p70 S6 Kinase Antibody (9202S) were purchased from Cell Signaling Technology. Furthermore, mToR (28273-1-AP) and GAPDH Polyclonal antibody (10494-1-AP) were obtained from Proteintech.

### 2.2. Animals and Treatments

In total, 45 male C57BL/6J mice, weighing 25 ± 2 g and 8 weeks old, were acquired from Beijing HFK Bioscience Co., Ltd. (Beijing, China). All mice were housed using a 12-h light/dark cycle in environments free of specific pathogens at 25 ± 1 °C and relative humidity of 55–60%. All mice also had unrestricted access to food and drink. According to the US National Institutes of Health and Public Health’s “Guide for the Care and Use of Laboratory Animals”, all experimental animal operations were carried out according to guidelines. Additionally, the Committee on the Ethics of Animal Experiments of China Agricultural University also reviewed and approved the animal care and experimental protocols (Approval No.: AW60212202-5-1).

After 7 days of acclimation, mice were randomly assigned to three groups with 15 animals per group: Control (CK), D-gal and D-gal + Nob groups. As previously published, D-gal was subcutaneously injected into 8-week-old C57BL/6J mice to create a model of skeletal muscle atrophy [20]. Except for the CK group, the mice in the other two groups received daily D-gal injections at a dose of 500 mg/kg body weight for 10 weeks. In contrast, the mice in the CK group received the same amount of 0.9% saline. Meanwhile, the D-gal + Nob group’s mice were administered an oral gavage of Nob at 100 mg/kg/day body weight following a D-gal injection. Moreover, the other two groups of mice were administered the same volume of 0.9% saline (containing DMSO). Every week, the body weights of every mouse were measured. Following blood sample collection via retro-orbital puncture on the last day of the experiment, all mice were cervically dislocated and executed on the last day of the experiment. Furthermore, muscle samples were collected, snap-frozen in liquid nitrogen and then transferred to −80 °C for later analysis.

### 2.3. Body Composition

After the end of the experiments, body mass composition was examined by nuclear magnetic resonance, with reference to Sun’s method using the minispec mq NMR spectrometer [21]. The lean mass was automatically calculated from the time-domain [^1^H] NMR signal through the instrument software and expressed in grams and percentages.

### 2.4. Muscle Fatigue Test

Muscular endurance test was performed by the motorized treadmill. After 3 days of acclimation, the mice were placed on the treadmill with the speed of 6 m/min. The running speed were increased every 5 min with 1 m/min until the mice showed fatigue defined by an inability to return to the treadmill or staying on the electrical shock grids for 10 s.

### 2.5. Grip Strength Test

Grip strength test was performed by using the GripTest instrument. Each animal was subjected to five trials each for forelimb and hindlimb, and average values were calculated.

### 2.6. Histological Staining

Isolated tibialis anterior (TA) muscle was fixed in 4% paraformaldehyde/PBS for a week at 4 °C followed by replacement with 70% EtOH and maintained at 4 °C minimum overnight. Tissues were processed with paraffin embedding, sectioning, and placed on slides. Dewaxed sections were processed with regular hematoxylin and eosin staining (HE). The mean muscle myofibers’ cross-sectional area and the myofiber number of the maximum cross-sectional area of TA tissue were counted through ImageJ software V1.8.0.112.

### 2.7. Transmission Electron Micrographs (TEMs)

The TA muscle samples were processed as follows: cut into small pieces of 3 × 1 × 1 mm along the direction of muscle fiber, fixed with 2.5% glutaraldehyde solution at 4 °C away from light for one week, fixed with osmic acid, dehydrated with 50%, 70%, 80%, 90% and 100% ethanol in turn, and then embedded with 1:1 acetone resin. After 4–5 h, the sample was transferred to the pure embedding solution, which could last overnight at room temperature. Subsequently, the embedded block was trimmed and ultrathin sectioned, and the muscle fibers were observed under the transmission electron microscope with a magnification of 2000 times. 

### 2.8. Extraction of Myofibrillar Protein

Myofibrillar protein was extracted from TA muscle using the Wang et al. method [22]. In brief, we collected 1 g of TA muscle, added four volumes (*v*/*w*) of salt solution (10 mmol/L sodium phosphate buffer, 2 mmol/L MgCl_2_, 100 mmol/L NaCl, 1 mmol/L EGTA, pH 7.0), homogenized on ice, and centrifuged at 4 °C for 15 min (2000× *g*). After discarding the supernatant, we repeated the above steps twice. The pellet was washed twice with 100 mmol/L NaCl, and the myofibril suspension was filtered with a 100-mesh filter cloth to remove connective tissue before the last centrifugation. Myofibrillar protein concentrations were determined by the BCA Protein Assay Kit.

### 2.9. Sodium Dodecyl Sulfate-Polyacrylamide Gel Electrophoresis (SDS-PAGE)

SDS-PAGE was performed following the published Wang et al. method [22]. Myofibrillar protein was mixed (4:1, *v*/*v*) with 5× SDS-PAGE protein loading buffer. After heating at 95 °C for 5 min, samples were placed at −20 °C and used as soon as possible. The changes in myofibrillar proteins in the CK, D-gal, and D-gal + Nob groups were analyzed using 12% separation gel and 4% concentrated gel.

### 2.10. RNA Extraction and Real-Time qPCR (RT-qPCR)

Total RNA was isolated from TA muscle using the TRIzol method (Invitrogen, Thermo Fisher Scientific, Waltham, MA, USA). Two micrograms of the isolated RNA were then used to synthesize cDNA. Finally, RT-qPCR was performed using the synthesized cDNA. The primer sequences are listed in Table 1.

### 2.11. Protein Extraction and Western Blotting

Western blotting and protein sample preparation were performed according to. [21]. We harvested 50 mg of TA muscles and homogenized it in 500 μL RIPA buffer (containing 100 mM PMSF), centrifuged at 4 °C for 15 min (12,000× *g*), and collected the supernatant and then used the BCA Protein Assay Kit to determine the protein concentration. The protein samples were mixed (4:1, *v*/*v*) with a 5× sample loading buffer. After boiling at 95 °C for 5 min, the samples were placed at −80 °C. 

### 2.12. Determination of Inflammatory Cytokines

The collected blood was stored at room temperature for ~2 h and then centrifuged at 4 °C for 15 min (3500 rpm). The blood supernatant was collected and transferred to −80 °C for subsequent analysis. We measured serum inflammation markers (LBP, TNF-α, and IL-6) according to the ELISA kit’s instructions.

### 2.13. Statistical Analysis

All data are shown as mean ± SEM. Differences between the two groups were analyzed using one-way ANOVA followed by Duncan’s post hoc test. *p*-value < 0.05 was considered to be statistically significant. 

## 3. Results

### 3.1. Effect of Nobiletin (Nob) on Skeletal Muscle Mass and Function in D-Galactose-Induced (D-Gal-Induced) Aging Mice 

**Figure 1:**
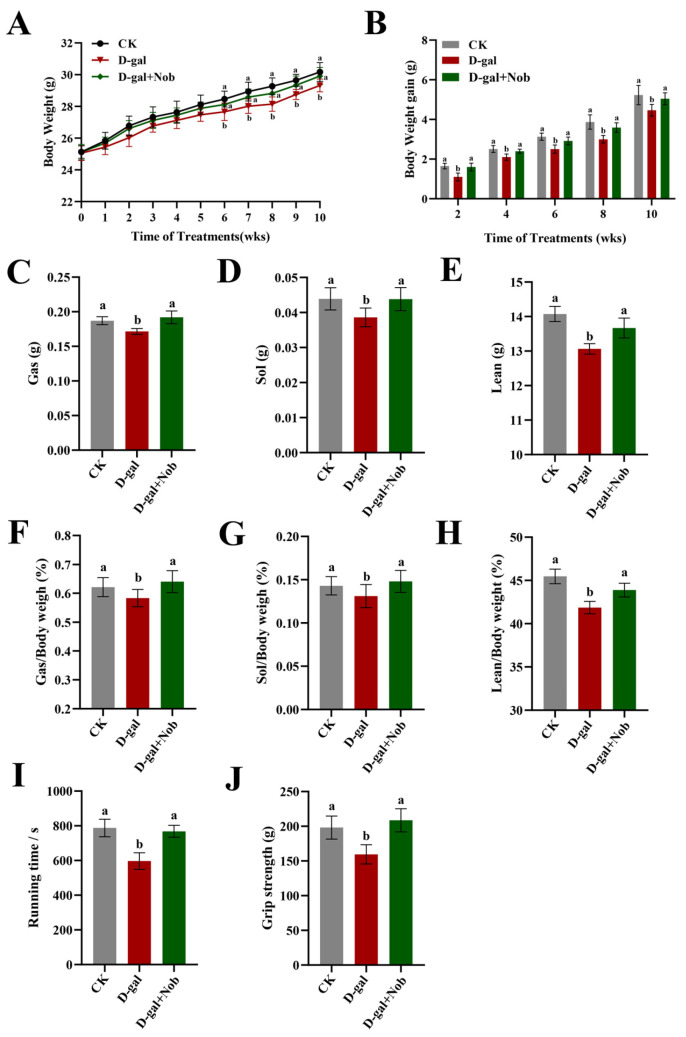
Nob increased skeletal muscle mass in D-gal-induced aging mice. (**A**,**B**) Body weight (BW) (**A**) and BW gain (**B**) in the CK, D-gal, and D-gal + Nob groups, *n* = 10. (**C**,**D**) The weight of gastrocnemius (Gas) and Gas to BW in the CK, D-gal, and D-gal + Nob groups, *n* = 10. (**E**,**F**) The weight of soleus (Sol) and Sol to BW in the CK, D-gal, and D-gal + Nob groups, *n* = 10. (**G**,**H**) The weight of lean and lean to BW in the CK, D-gal, and D-gal + Nob groups, *n* = 8. (**I**,**J**) Running time and grip strength in the CK, D-gal, and D-gal + Nob groups, *n* = 6. Significant differences between treatment groups are represented by the lowercase letters a and b (*p* < 0.05).

Using D-gal-induced C57BL/6J mice to imitate the skeletal muscle atrophy during aging process, we investigated the effect of Nob on sarcopenia [6]. All mice showed BW gain at 10 weeks. The D-gal-induced mice showed markedly lower BW and BW gain than the CK mice after receiving a D-gal injection for 6 weeks (*p* < 0.05) (Figure 1A,B). Nob partially returned the BW and BW gain of the D-gal-induced mice to normal levels compared to the CK group (Figure 1A,B). In addition, the weight of Gas and Sol (the ratio of Gas/BW and Sol/BW) in the D-gal mice was greatly reduced relative to the CK mice, yet partially restored by Nob therapy (*p* < 0.05) (Figure 1C–F). Consistent with its effect on BW, body mass composition analysis revealed that Nob significantly increased lean weight (the ratio of lean/BW) in the D-gal mice compared with the CK mice (*p* < 0.05). Notably, we also determined that Nob markedly improved muscular endurance (running time) and grip strength in D-gal-induced mice (*p* < 0.05). Therefore, Nob has a good antagonistic effect on the skeletal muscle mass and function loss of the D-gal-induced mice.

### 3.2. Effect of Nob on the Number and Structure of Myofiber in D-Gal-Induced Aging Mice

**Figure 2:**
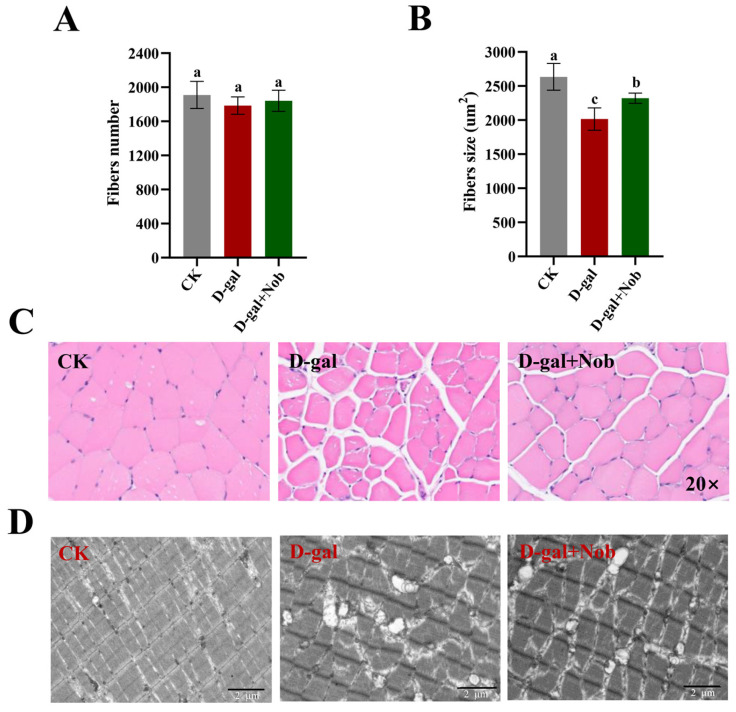
Nob improved skeletal muscle atrophy in D-gal-induced aging mice. (**A**) The number of myofibers in the CK, D-gal, and D-gal + Nob groups, *n* = 5. (**B**)The myofiber size (mean cross-section area) in the CK, D-gal, and D-gal + Nob groups, *n* = 5. (**C**) Representative images of hematoxylin and eosin (HE) staining of myofiber in the CK, D-gal, and D-gal + Nob groups (Magnification ×20), *n* = 5. (**D**) Representative images of myofiber ultrastructure in the CK, D-gal, and D-gal + Nob groups. Scale bar, 2 μm, *n* = 3. Significant differences between treatment groups are represented by the lowercase letters a, b, and c (*p* < 0.05).

Next, we aim to examine Nob’s effect on myofiber’s number and structure in the CK, D-gal, and D-gal + Nob groups. We identified that myofiber numbers were not significantly altered between different treatment groups (Figure 2A). However, HE staining demonstrated that the myofiber size in the D-gal-induced mice was significantly decreased relative to the CK mice (*p* < 0.05). Additionally, Nob intervention significantly improved myofiber size in D-gal-induced mice (*p* < 0.05) (Figure 2B,C). The ultrastructural analysis of myofibers showed that in the CK group, the structure of myofibers was complete and arranged in a tight and orderly manner. Notably, the bright band (I band), dark band (A band), Z line, and M line were clearly and orderly arranged. In the D-gal group, the structure of myofibers was completely disordered, and the I band, A band, Z line, and M line disappeared, and the thick and thin filaments were loosely arranged. Myofiber structure was complete in the D-gal + Nob mice, and the organization of the I band, A band, Z line, and M line was obvious (Figure 2D).

### 3.3. Effect of Nob on Skeletal Muscle Composition in D-Gal-Induced Aging Mice

**Figure 3:**
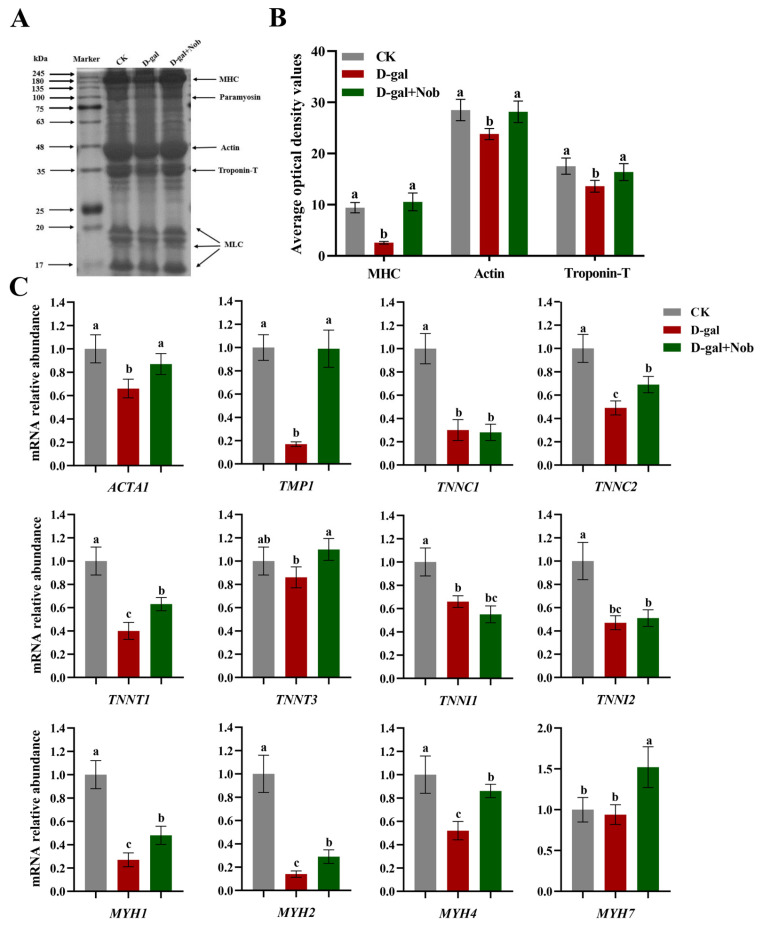
Nob improved skeletal muscle composition in D-gal-induced aging mice. (**A**,**B**) SDS-PAGE images and relative abundance of myofibrillar protein in the CK, D-gal and D-gal + Nob groups, *n* = 3. (**C**) mRNA of *ACTA1*, *TMP1*, *TNNC1*, *TNNC2*, *TNNT1*, *TNNT3*, *TNNI1*, *TNNI2*, *MYH1*, *MYH2*, *MYH4,* and *MYH7* in the CK, D-gal and D-gal + Nob groups, *n* = 3. Significant differences between treatment groups are represented by the lowercase letters a, b, and c (*p* < 0.05).

Changes in protein mass and integrity affect muscle physiology, quality, and function [5]. Myofibrillar protein is the main component of skeletal muscle, making up about 55–60% of skeletal muscle [23]. Thus, we next conducted the SDS-PAGE analysis on myofibrillar protein in skeletal muscle and determined that myosin heavy chain, tropomyosin, actin, and troponin T in D-gal-induced aging mice decreased to varying degrees. Likewise, the expression of these proteins was also shown to be dramatically increased by Nob intervention (Figure 3A,B). In addition, analysis by RT-qPCR revealed that D-gal induction could significantly reduce the expression of actin (*ACTA1*), tropomyosin (*TMP1*), troponin complex (*TNNC1*, *TNNC2*, *TNNT1*, *TNNI1* and *TNNI2*) and myosin heavy chain (*MYH1*, *MYH2* and *MYH4*) relative to the CK mice. Furthermore, Nob intervention partly restored the mRNA expression of actin (*ACTA1*), tropomyosin (*TMP1*), troponin complex (*TNNC2*, *TNNT1* and *TNNT3*), and myosin heavy chain (*MYH1*, *MYH2* and *MYH4*) to the CK levels (Figure 3C). 

### 3.4. Effect of Nob on Skeletal Muscle Protein Synthesis in D-Gal-Induced Aging Mice

**Figure 4:**
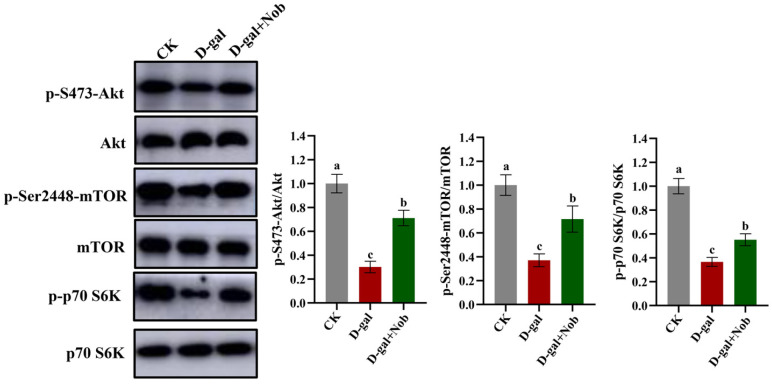
Nob promoted skeletal muscle protein synthesis in D-gal-induced aging mice. Western blot analysis of Akt, p-S473-Akt, mTOR, p-Ser2448-mTOR, p70 S6K, p-p70 S6K and GAPDH in the CK, D-gal and D-gal + Nob groups, *n* = 3. Significant differences between treatment groups are represented by the lowercase letters a, b, and c (*p* < 0.05).

To further understand the underlying molecular mechanism of Nob in the treatment and prevention of skeletal muscle atrophy, we examined the protein synthesis signaling pathways (mTOR/Akt) in the CK, D-gal, and D-gal + Nob groups. As shown in Figure 4, compared with the CK group, the ratio of p-S473-Akt/Akt and p-Ser2448-mTOR/mTOR marked a decrease in the D-gal-induced mice, illustrating that mTOR/AKT pathway was inhibited to reduce protein synthesis. However, Nob intervention partly returned the ratio of p-S473-Akt/Akt and p-Ser2448-mTOR/mTOR proteins to the CK group-levels (Figure 4). Additionally, Nob therapy activated the downstream target p-p70 S6K phosphorylation in the D-gal-induced mice, indicating enhanced protein synthesis (Figure 4) [3]. Thus, these data showed that Nob could act on aging skeletal muscle to activate mTOR/Akt signaling and enhance protein synthesis.

### 3.5. Effect of Nob on Skeletal Muscle Protein Degradation in D-Gal-Induced Aging Mice

**Figure 5:**
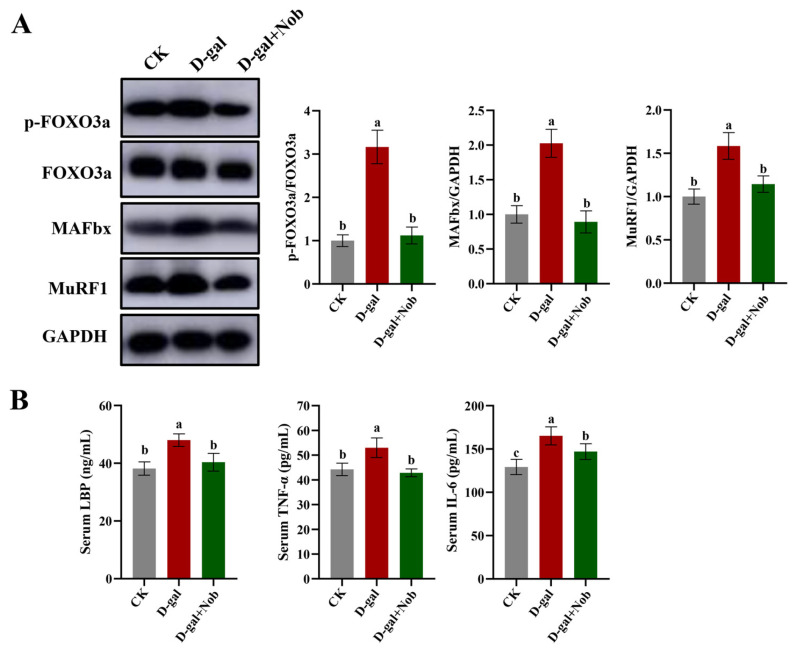
Nob-inhibited skeletal muscle protein degradation in D-gal-induced aging mice. (**A**) Western blot analysis of FoxO 3a, p-FoxO 3a, MAFbx, MuRF1, and GAPDH in the CK, D-gal and D-gal + Nob groups, *n* = 3. (**B**) Inflammation markers including LBP, TNF-α, and 1L-6 in the CK, D-gal and D-gal + Nob groups, *n* = 8. Significant differences between treatment groups are represented by the lowercase letters a, b, and c (*p* < 0.05).

We next detected protein degradation pathways in the CK, D-gal, and D-gal + Nob groups. The transcription factors FoxO, negatively regulated by the IGF1-Akt pathway, and NF-ĸB, activated by inflammatory factors, are critical to skeletal muscle atrophy [24]. In our research, the ratio of p-FOXO 3a/FOXO was significantly increased in the D-gal-induced mice relative to the CK mice (*p* < 0.05) (Figure 5). Likewise, compared to CK mice, D-gal-induced mice had significantly higher levels of MAFbx and MuRF1 expression (*p* < 0.05). Importantly, Nob intervention severely reduced MAFbx and MuRF1 expression (Figure 5). Finally, we examined inflammation indicators at serum. We discovered that Nob markedly reduced D-gal-induced increases in the levels of lipopolysaccharide-binding peptide (LBP), tumor necrosis factor-α (TNF-α), and interleukin-6 (IL-6) (Figure 5). Therefore, these findings show that Nob has a strong inhibitory effect against skeletal muscle protein breakdown in D-gal-induced aging animals.

## 4. Discussion

Sarcopenia decreases skeletal muscle mass and function caused by aging, impairs mobility, raises the risk of fractures, diabetes, and other illnesses, and severely affects a senior’s quality of life [25]. However, no effective drugs are approved for clinical use to prevent or treat sarcopenia, and its healthcare burden remains high [26,27]. Given the world’s aging population, finding safe and efficient food-derived substances to counteract sarcopenia is crucial. Thus, we investigated Nobiletin (Nob), a natural polymethoxyl flavonoid extracted from citrus peels [11]. This study reported that Nob could increase body weight, gastrocnemius (Gas) mass, soleus (Sol) mass, lean mass and improve skeletal muscle function in D-galactose-induced (D-gal-induced) mice. Additionally, Nob improved myofiber size and increased major constituent proteins of skeletal muscle, such as myosin heavy chain, actin, troponin complex, etc. The present research also discovered that Nob could maintain protein homeostasis to prevent skeletal muscle atrophy. Therefore, these findings provide strong evidence for Nob to treat and prevent the loss of skeletal muscle.

The D-gal-induced aging model has been extensively used in aging and anti-aging research [28]. Notably, D-gal accelerated aging model is based on the metabolic theory and has been widely applied to in vitro and in vivo studies due to its convenience, minimal side effects, high survival rate, and similar performance to the natural aging process in many ways [28]. Likewise, it is well known that D-gal-induced oxidative stress and inflammation could lead to skeletal muscle atrophy [6,29,30,31]. In our work, we established a model of sarcopenia using subcutaneous administration of 500 mg/kg/day of D-gal to C57BL/6J mice for 10 weeks, according to the methods of Gao’s study [32]. Compared with the CK mice, 10-week D-gal induction significantly reduced muscle mass (including Gas and Sol mass) and myofiber size, consistent with Wu et al.’s and Kou et al.’s findings [6,30]. Thus, these findings indicate that D-gal could induce skeletal muscle atrophy. In addition, we used 100 mg/kg/day of Nob to interfere with D-gal-induced mice according to the previous studies of Nohara et al. and He et al. [12,33]. We determined that the Nob intervention could partly restore these parameters to the CK levels, indicating that Nob could prevent skeletal muscle atrophy.

Myofibrillar proteins comprise 55–60% of muscle protein and are the structural proteins that consist of the myofibrils [22]. Among them, the most abundant myofibrillar is myosin (500 kDa), which accounts for ~43%. Notably, myosin consists of two MHC (myosin heavy chains, 220 kDa) and four MLC (myosin light chains, 16–25 kDa) [22]. Additionally, actin (42 kDa) is the main component of the thin filament of the myofibrils, accounting for about 22% of myofibrillar protein. Importantly, myosin and actin are directly responsible for muscle contraction and are the backbone of myofibrils [22]. Tropomyosin (70 kDa) is also composed of thin filaments of myofibrils and is the most abundant regulatory protein, accounting for about 8% of myofibrillar protein [22]. Likewise, the troponin complex is a component of the thin filament of myofibrils, including troponin C, troponin I and troponin T (respectively, 18 kDa, 21 kDa, and 35 kDa), accounting for about 5% of muscle fibers [22]. Tropomyosin and troponin complexes mainly initiate and control muscle contraction [22]. Consequently, proteolytic systems are activated during skeletal muscle atrophy, removing damaged organelles and contractile proteins, which cause myofibers to atrophy [16]. In our study, we conducted the SDS-PAGE analysis on myofibrillar protein in skeletal muscle. Compared to the CK mice, D-gal-induced mice demonstrated lower levels of the myosin heavy chain, tropomyosin, actin, and troponin T proteins. In contrast, Nob partially recovered these proteins to the CK mice levels (Figure 3A,B). Likewise, D-gal induction significantly reduced actin (*ACTA1*), tropomyosin (*TMP1*), troponin complex (*TNNC1*, *TNNC2*, *TNNT1*, *TNNI1*, *TNNI2*) and myosin heavy chain (*MYH1*, *MYH2*, *MYH4*) mRNA expression compared to the CK mice. Conversely, Nob intervention strongly reversed the D-gal-induced changes (Figure 3C). Thus, these data suggested Nob could tilt the balance of protein homeostasis. 

Protein homeostasis balances protein synthesis and breakdown processes to adjust myofiber size in reaction to anabolic and stress signals [5,23]. Thus, we subsequently examined the protein synthesis (mTOR/Akt) and protein degradation (UPP) signal pathways. Notably, previous studies reported that protein homeostasis was disrupted during aging [5], in which the mTOR/AKT pathway is a critical regulatory pathway of protein homeostasis [5]. Likewise, Akt could regulate both mTOR-mediated protein synthesis and FoxO-mediated protein breakdown [16]. Our study reported that Nob can act on D-gal-induced aging skeletal muscle to activate the mTOR/Akt signaling and further activate the downstream target p70 S6K phosphorylation, indicative of increased protein synthesis (Figure 4) [3]. In addition, the present study reported that Nob could improve skeletal muscle atrophy by activating Akt to block FOXO 3a-MAFbx/MuRF1 signaling and inhibit inflammatory cytokines to subsequently reduce protein degradation. Notably, some studies discovered that the expression of the E3 ubiquitin ligases MAFbx and MuRF1 was increased in aged rat and human muscles [34,35,36], similar to our findings in D-gal-induced muscles. Therefore, these data suggest a powerful efficacy of Nob to counteract skeletal muscle atrophy.

## 5. Conclusions

**Figure 6:**
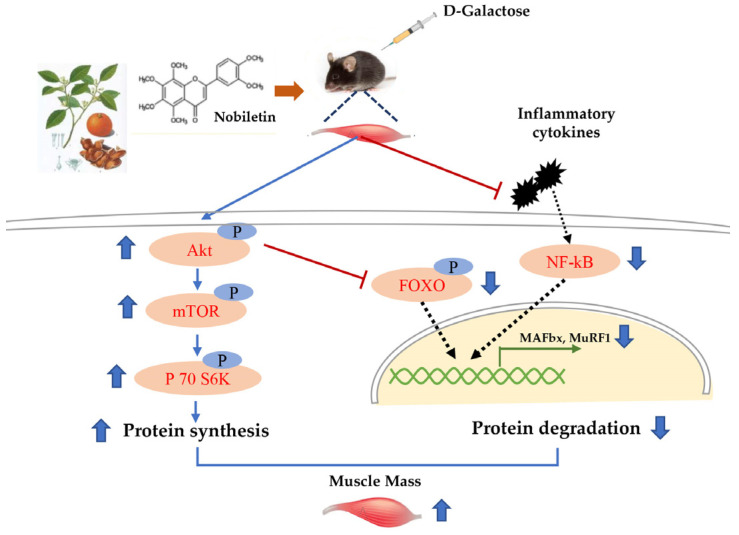
Schematic diagram of Nob-mediated prevention of skeletal muscle atrophy. Protein kinase B (AKT); Rapamycin (mTOR); Ribosomal protein S6 kinase beta-1 (S6K1), also called P 70 S6 kinase (P 70 S6K), Nuclear factor-kappa B (NF-ĸB), Foxhead box O3 (FOXO3), Muscle atrophy F-box (MAFbx), Muscle RING Finger 1 (MuRF1).

Supplementing Nob could increase body weight, hindlimb muscle mass, lean mass and improved skeletal muscle function. In addition, Nob could improve myofiber size and skeletal muscle main protein composition in D-gal-induced aging mice. Furthermore, Nob treatment activated the mTOR/Akt signaling to increase protein synthesis and inhibit FoxO3a-MAFbx/MuRF1 pathway and inflammatory cytokines (LBP, TNF-α and 1L-6), thereby reducing protein degradation (Figure 6). Therefore, Nob regulates protein homeostasis to ameliorate D-gal-induced skeletal muscle atrophy and may be a promising candidate for clinical treatment and prevention of age-associated muscle loss.

## Figures and Tables

**Table 1:** List of primer sequences for RT-qPCR.

Gene	Sequence 5′ → 3′ (Forward)	Sequence 5′ → 3′ (Reverse)
*ACTA1*	TACCACCGGCATCGTGTTG	GCGCACAATCTCACGTTCAG
*TMP1*	TTGAAAGCCGAGCCCAAAAAG	TCATACTTCCGGTCAGCATCTT
*TNNC1*	GCGGTAGAACAGTTGACAGAG	GACAGAAACTCATCGAAGTCCA
*TNNC2*	GAGGCCAGGTCCTACCTCAG	GGTGCCCAACTCTTTAACGCT
*TNNT1*	ACTAAAAGACCGCATTGGAGG	AGCTCCCATGTTGGACAGAAC
*TNNT3*	ACTGCTCCTAAGATCCCGGAA	ATGAGGTCCTTGTTTTGACGC
*TNNI1*	ATGCCGGAAGTTGAGAGGAAA	TCCGAGAGGTAACGCACCTT
*TNNI2*	CGGAGGGTGCGTATGTCTG	CAGGTCCCGTTCCTTCTCA
*MYH1*	CTCTTCCCGCTTTGGTAAGTT	CAGGACATTTCGATTAGATCCG
*MYH2*	TGGAGGGTGAGGTAGAGAGTG	TTGGATAGATTTGTGTTGGATTG
*MYH4*	CCGCATCTGTAGGAAGGGG	GTGACCGAATTTGTACTGATGT
*MYH7*	CCTGCGGAAGTCTGAGAAGG	CTCGGGACACGATCTTGGC
*GAPDH*	TGGCCTTCCGTGTTCCTAC	GAGGCTGTGAAGTCGCA

## Data Availability

The data presented in this study are available in the article.
